# Short-Course Radiotherapy Followed by Neoadjuvant Bevacizumab, Capecitabine, and Oxaliplatin and Subsequent Radical Treatment in Primary Stage IV Rectal Cancer: Long-Term Results of a Phase II Study

**DOI:** 10.1245/s10434-017-5897-0

**Published:** 2017-05-30

**Authors:** C. Bisschop, T. H. van Dijk, J. C. Beukema, R. L. H. Jansen, H. Gelderblom, K. P. de Jong, H. J. T. Rutten, C. J. H. van de Velde, T. Wiggers, K. Havenga, G. A. P. Hospers

**Affiliations:** 1 Department of Medical Oncology, University of Groningen, University Medical Center Groningen, Groningen, The Netherlands; 2 Department of Surgery, University of Groningen, University Medical Center Groningen, Groningen, The Netherlands; 3 Department of Radiation Oncology, University of Groningen, University Medical Center Groningen, Groningen, The Netherlands; 4 Department of Medical Oncology, Maastricht University Medical Center, Maastricht, The Netherlands; 5 Department of Medical Oncology, Leiden University Medical Center, Leiden, The Netherlands; 6 Department of Hepato-Pancreato-Biliary Surgery, University of Groningen, University Medical Center Groningen, Groningen, The Netherlands; 7 Department of Surgery, Catharina Hospital, Eindhoven, The Netherlands; 8 GROW: School of Oncology and Developmental Biology, University of Maastricht, Maastricht, The Netherlands; 9 Department of Surgery, Leiden University Medical Center, Leiden, The Netherlands

## Abstract

**Background:**

In a Dutch phase II trial conducted between 2006 and 2010, short-course radiotherapy followed by systemic therapy with capecitabine, oxaliplatin, and bevacizumab as neoadjuvant treatment and subsequent radical surgical treatment of primary tumor and metastatic sites was evaluated. In this study, we report the long-term results after a minimum follow-up of 6 years.

**Methods:**

Patients with histologically confirmed rectal adenocarcinoma with potentially resectable or ablatable metastases in liver or lungs were eligible. Follow-up data were collected for all patients enrolled in the trial. Overall and recurrence-free survival were calculated using the Kaplan–Meier method.

**Results:**

Follow-up data were available for all 50 patients. After a median follow-up time of 8.1 years (range 6.0–9.8), 16 patients (32.0%) were still alive and 14 (28%) were disease-free. The median overall survival was 3.8 years (range 0.5–9.4). From the 36 patients who received radical treatment, two (5.6%) had a local recurrence and 29 (80.6%) had a distant recurrence.

**Conclusions:**

Long-term survival can be achieved in patients with primary metastatic rectal cancer after neoadjuvant radio- and chemotherapy. Despite a high number of recurrences, 32% of patients were alive after a median follow-up time of 8.1 years.

In approximately 20% of rectal cancer patients, the tumor is already metastasized at first presentation.^1^,^2^ These patients have a poor prognosis, with a life expectancy of 1 year after diagnosis.^3^ Presentation with synchronous metastases creates a treatment dilemma: which tumor location poses the biggest threat, and should treatment initially be directed towards the primary tumor or the distant metastases.

In rectal cancer with synchronous metastases, the primary tumor has usually already progressed to a more locally advanced stage (T3 or T4 according to the American Joint Committee on Cancer [AJCC] staging system), with or without involvement of locoregional lymph nodes. Advanced primary tumors require downstaging to enable resection with adequate margins. Downstaging can be accomplished with neoadjuvant radiotherapy or chemoradiotherapy.^4^,^5^ Long-course radiotherapy with concomitant 5-fluorouracil-based chemotherapy is the standard neoadjuvant regimen for locally advanced rectal cancer.^5^ In patients with synchronously metastasized rectal cancer, this neoadjuvant scheme creates a long interval between the start of treatment and surgery during which distant metastases may progress. Furthermore, 5-fluorouracil, or its oral analog capecitabine, acts as a radiosensitizer and has no or little effect on distant metastases.

In a Dutch trial, known in The Netherlands as the M1 study, a new chemoradiotherapy regimen was proposed for patients with rectal cancer and synchronous metastases.^6^ In this study, patients were treated with a short-course of radiotherapy (5 × 5 Gy), followed by chemotherapy that consisted of a combination of capecitabine, oxaliplatin and bevacizumab, and eventually followed by radical treatment of all tumor sites. In this regimen, short-course radiotherapy is administered to achieve control of the primary tumor, while systemic treatment with capecitabine and oxaliplatin (CapeOx) in combination with bevacizumab is administrated as treatment for metastatic disease.^7^,^8^ In 72% of participants, radical treatment of both metastases and the primary tumor could be performed. In the initial analysis, patients had been followed for at least 2 years. The aim of this update is to study the impact of the study treatment on long-term survival and recurrence of disease.

## Materials and Methods

### Study Design

Full details of the design, patients’ eligibility criteria, and procedures have been reported previously.^6^ In brief, this study is an open-label, phase II clinical study that is registered with the Netherlands Trial Register (NTR2029). Between April 2006 and December 2010, 50 patients were included in seven centers in The Netherlands. The primary endpoint of the initial study was the percentage of patients receiving radical treatment of all tumor sites. As secondary endpoints, 2-year survival, 2-year local and distant recurrence rate and treatment-related toxicity were assessed.

### Eligibility

Patients were eligible for this study if they were diagnosed with primary metastasized rectal cancer and were at least 18 years of age. Rectal adenocarcinoma should be histologically confirmed, and liver or lung metastases should be eligible for resection or radiofrequency ablation (RFA). The Institutional Review Board at each participating center approved the study protocol, and all patients provided written informed consent.

### Treatment and Study-Related Procedures

Treatment and procedures have been described previously.^6^ In summary, preoperative treatment started with external beam radiotherapy (5 × 5 Gy) on the mesorectum and regional lymph nodes. The upper field border was set at L5/S1. Within 2 weeks after completion of radiotherapy, chemotherapy started with six cycles of 3 weeks, consisting of capecitabine-oxaliplatin-bevacizumab. On the first day of a chemotherapy cycle, patients received bevacizumab (7.5 mg/kg) and oxaliplatin (130 mg/m^2^), both as an intravenous infusion. During the first 2 weeks of each cycle, capecitabine (1000 mg/m^2^) was administered as an oral drug. Surgery was planned 6–8 weeks after the last dose of bevacizumab, and the surgical treatment strategy was personalized for each patient by a multidisciplinary team. Radical treatment (R0) of the liver metastases was defined as microscopic tumor-free resection margins or an ablation zone completely covering the metastasis, including a ≥5 mm peritumoral margin on the 1-week post-RFA computed tomography (CT) scan.

Baseline imaging of the primary tumor was performed with a pelvic contrast-enhanced CT or magnetic resonance imaging (MRI), and, for imaging of distant metastases, a CT scan of the chest and abdomen was used. Radiologic response evaluation was performed after two cycles of chemotherapy using a CT scan. If no progression of disease was seen on the CT scan, patients additionally received four cycles of chemotherapy. After completion of preoperative treatment, resectability of both the primary tumor and metastases were reassessed using the same imaging procedures as were used at baseline.

Histopathologic evaluation of the resection specimens was performed by a pathologist following local standards, and pathologic response after neoadjuvant therapy was assessed using Mandard’s classification.^9^

### Survival Follow-Up and Statistics

Follow-up visits as part of the study were carried out every 3 months during the first 3 years. Thereafter, follow-up was at the physician’s discretion. Overall and recurrence-free survival were calculated using the Kaplan–Meier survival analysis method, with overall survival being defined as the time from the start of radiotherapy until death from any cause. Recurrence-free survival was calculated for all patients in whom radical surgery was possible, with recurrence-free survival being defined as the time from radical surgery (after which the patient was rendered disease-free) to the diagnosis of first recurrence. The log-rank test was used to compare the survival distribution of subgroups. All statistical analyses were performed using SPSS (Released 2013. IBM SPSS Statistics for Windows, Version 22.0; IBM Corporation, Armonk, NY, USA), and a *p* value of ≤0.05 was regarded as statistically significant.

## Results

### Patient Characteristics

Follow-up data were available for all 50 patients. Patient and treatment characteristics are described in Table 1. Median follow-up duration was 8.1 years (range 6.0–9.8). Thirty-six patients in the study received radical treatment (R0), 10 patients had irresectable disease after neoadjuvant radio- and chemotherapy, and in 4 patients the resection of the primary rectal tumor was not radical (R1). Eleven of the 43 patients (25.6%) who received a resection of the primary rectal tumor, showed a pathological complete response, and seven patients (16.3%) showed a pathological near-complete response of the primary tumor.

**Table 1:** Patient and tumor characteristics

Characteristics	Results [*N* = 50]
Age at start of treatment, years [median (range)]	59 (35–75)
Male sex	27 (54)
Clinical tumor category	
T2N0	0 (0)
T2N1	4 (8)
T3N0	6 (12)
T3N1-2	32 (64)
T4N0	1 (2)
T4N1-2	7 (14)
Metastatic site	
Liver	42 (84)
Lung	5 (10)
Lung and liver	3 (6)
Liver metastases	
1–3	36 (72)
>3	9 (18)
Lung metastases	
1	5 (10)
>1	3 (6)
Radiotherapy	
Patients completing radiotherapy	50 (100)
Interval between completion of RT and start of CT—days	
Median (range)	11 (3–44)
Chemotherapy	
Patients receiving six cycles	42 (84)
Surgery	
Radical surgical treatment	36 (72)
Sequence of surgery	
Simultaneous resection	25 (50.0)
Primary first	10 (20)
Metastases first	5 (10)

Data are expressed as *n* (%) unless otherwise specified

*RT* radiotherapy, *CT* chemotherapy

### Overall Survival

**Fig. 1:**
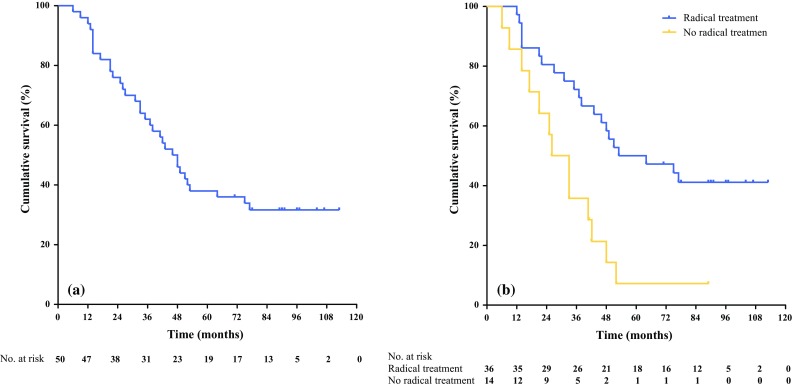
Overall survival (months) in **a** the total study population (*N* = 50) and **b** subgroups that did (*N* = 36) and did not (*N* = 14) receive radical treatment of primary tumor and/or distant metastases

Median overall survival of the total cohort was 3.8 years (range 0.5–9.4) (Fig. 1a). The overall survival rate after 2 years of treatment was 74% (37 of 50 patients). The 5-year survival rate was 38.0% (19 of 50 patients), and, after a median follow-up time of 8.1 years, 32.0% (16 of 50 patients) of patients were alive. Fourteen of these 16 patients (28%) were disease-free after 8.1 years of follow-up. In 36 of 50 patients, in whom radical (R0) treatment of primary tumor and metastatic sites was achieved, median overall survival was 4.4 years (Fig. 1b). Survival of these patients was significantly better than in patients in whom treatment was not radical (median overall survival 2.8 years; log-rank test *p* = 0.004).

### Recurrence Rate

**Fig. 2:**
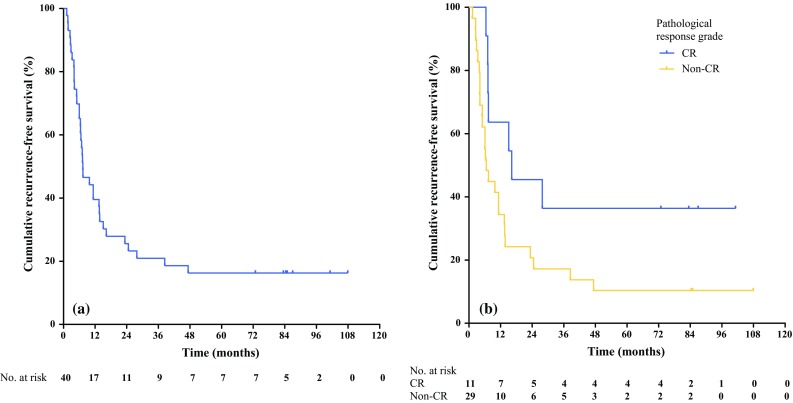
Recurrence-free survival (months) for **a** patients who received surgical treatment of all tumor sites (*N* = 40) and **b** subgroups with (*N* = 11) or without (*N* = 29) pathological complete response after surgical treatment in the study. *CR* complete response

Of the 36 patients who received radical treatment, 29 (80.6%) had recurrence of the disease, with most of these recurrences being distant metastases. Only two patients developed a local recurrence, one in combination with a distant recurrence. Both patients with a local recurrence had a pathological partial response of the initial primary tumor. The number and location of recurrences are provided in Table 2. The 2- and 5-year recurrence rates were 72.2 and 80.6%, respectively. Patients with recurrence of metastatic disease had a median number of two metastases at presentation with recurrent disease. One of the patients with a local recurrence and 18 of 28 patients (64.3%) with a distant recurrence could be rendered disease-free after successful treatment of the recurrence. Treatment of the distant recurrences consisted of surgery (11), RFA of liver metastases (4), and stereotactic radiosurgery (3). Fourteen of these 18 patients (77.8%) developed a second recurrence. Median recurrence-free survival, calculated after the completion of treatment in this trial, was 7.4 months (Fig. 2a). Median time to recurrence was 7.1 months. Median recurrence-free survival for subgroups with or without pathological complete response was 16.2 vs. 6.6 months, and the difference between the groups was statistically significant (log-rank test *p* = 0.039) (Fig. 2b).

**Table 2:** Anatomic location of recurrences of rectal cancer in patients receiving radical treatment (*N* = 36)

Anatomic location	Frequency	Percentage
Rectum (local)	2	5.6
Liver	10	27.8
Lung	9	25.0
Liver and lung	3	8.3
Liver, lung, and lymph nodes	2	5.6
Other	4	11.1

**Fig. 3:**
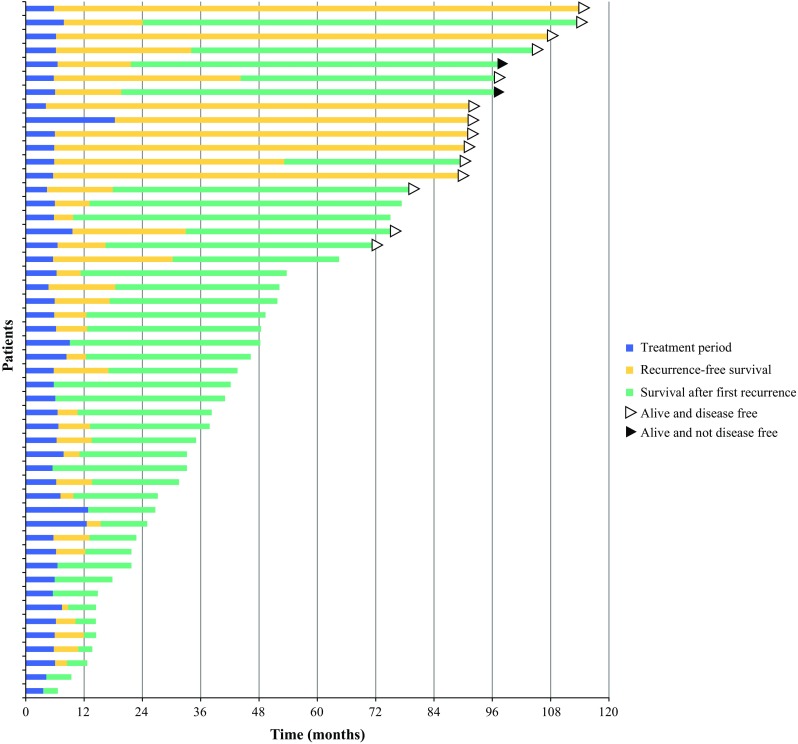
Swimmers plot illustrating overall survival of all individual patients in the study. Survival is divided into three periods: treatment phase, recurrence-free survival, and survival after first recurrence

Overall and recurrence-free survival of all individual patients are illustrated in a swimmers plot (Fig. 3).

## Discussion

Long-term follow-up of patients in this trial showed that long-term survival can be achieved in patients with primary metastatic rectal cancer with a treatment regimen consisting of neoadjuvant radio- and chemotherapy followed by surgical resection of both the primary tumor and metastatic sites. Thirty-two percent of patients were alive after a median follow-up of 8.1 years, and 28% of patients were disease-free at that time.

The number of long-term survivors in this trial can be considered high, regarding the poor prognostic features of the study population. For example, synchronous occurrence of distant metastases and an advance stage of the primary tumor are known as poor prognostic features in colorectal cancer.^10^ In addition, patients with metastases in multiple organs, who were eligible for this study, have a worse progression-free survival.^11^ The median overall survival of 3.8 years is longer than could be expected in patients with primary stage IV rectal cancer,^4^ but is in accordance with other studies investigating neoadjuvant treatment before surgical resection of all tumor sites;^12^–^15^ however, these studies had a retrospective design and only included patients with liver metastases.

Despite the high number of long-term survivors, many radically treated patients experienced recurrence of disease (80.6%). Distant recurrences were mainly seen, which is known as the preferred pattern of recurrence in primary metastatic rectal cancer.^16^ Most of the recurrences occurred shortly after study treatment; 21 of 29 (72.4%) recurrences occurred within 1 year. The high early recurrence rate could be the result of missed (micro-) metastases by CT scan, which was the only mandatory method of radiological evaluation in this trial. Particularly in relation to the detection of extrahepatic tumor recurrences, which were seen in 20 of 36 (55.6%) patients who received radical treatment in this study, a combined positron emission tomography (PET)-CT scan outperformed a conventional CT scan.^17^,^18^ Because PET-CT could detect extrahepatic tumor sites at an earlier stage, a more radical treatment of all tumor locations could be performed to prevent recurrences. The high recurrence rate had no significant effect on survival, which may be explained by the fact that many patients could be treated with curative intent for their recurrence. Repeat treatments of recurrent colorectal metastases is possible in approximately 50% of patients, and contributes to a prolonged survival.^19^ The high and early recurrence rate in our study is in accordance with other studies.^12^,^16^

The aim of the chemotherapy regimen in this study was to achieve both local control of the primary rectal tumor and adequate treatment of distant metastases. Therefore short-course radiotherapy (5 × 5 Gy) was followed by adequate systemic cycles of capecitabine, oxaliplatin and bevacizumab. Systemic chemotherapy is capable of downsizing metastatic lesions and can convert unresectable or borderline resectable metastases into resectable metastases.^20^,^21^ The high number of pathological complete (25.6%) or near-complete (16.3%) responses and low local recurrence rate (5.6%) in this study indicates local control can be achieved with the regimen used in this study. A study in patients with symptomatic primary rectal tumors and unresectable distant metastases showed that short-course radiotherapy and oxaliplatin-based chemotherapy can achieve local control, even in patients with near-obstructing primary tumors.^22^,^23^ This treatment regimen led to a favorable palliative effect in nearly two-thirds of patients, and palliative surgery was needed in only 20% of patients. An important predictor for pathological response of the primary tumor is the time between radiotherapy and surgery, a longer interval being associated with a higher rate of pathological response.^24^ In this study, an interval of approximately 25 weeks existed between short-course radiotherapy and surgery in patients receiving all six cycles of chemotherapy, which could explain the high pathological response rate.

Timing and sequence of surgical resection of a primary tumor with metastatic sites is still subject to debate; surgical treatment could be directed at the primary tumor first, at the metastases first (liver-first approach), or both primary tumor and metastases could be treated simultaneously. Because resection of the primary tumor has no impact on overall survival as opposed to resection of the metastasis, and the metastases can potentially give rise to further systemic spread of the disease, the latter should not be delayed and a metastasis-first or simultaneous approach are preferable.^13^,^15^ Furthermore, the complication rate of a simultaneous resection is not inferior to a staged resection, but total hospital stay is significantly shorter.^12^,^25^ Both resection of the primary rectal tumor and resection of hepatic or extrahepatic rectal cancer metastases can cause significant morbidity;^26^,^27^ therefore, radiological restaging after neoadjuvant therapy is important to make sure that all tumor locations are still resectable. A simultaneous resection of primary tumor and distant metastases was pursued in this study, although in 15 patients this was not feasible and a staged resection was performed.

## Conclusions

We showed that long-term survival, as well as local control of the rectal tumor, can be achieved in patients presenting with rectal cancer and synchronous metastases in liver or lungs by preoperative treatment with short-course radiotherapy and chemotherapy with capecitabine, oxaliplatin and bevacizumab, followed by radical treatment. Patients should be carefully selected by a multidisciplinary team for this treatment strategy, and resectability of all tumor locations should be properly assessed at the start of treatment and reassessed after completion of neoadjuvant treatment.
